# Overlap of Gastroesophageal Reflux Disease and Functional Dyspepsia and Yield of Esophagogastroduodenoscopy in Patients Clinically Fulfilling the Rome IV Criteria for Functional Dyspepsia

**DOI:** 10.3389/fmed.2022.910929

**Published:** 2022-06-15

**Authors:** Duc Trong Quach, Quoc Van Ha, Chuyen Thi-Ngoc Nguyen, Quang Dinh Le, Doan Thi-Nha Nguyen, Nhu Thi-Hanh Vu, Ngoc Le-Bich Dang, Nhan Quang Le

**Affiliations:** ^1^Department of Internal Medicine, University of Medicine and Pharmacy at Ho Chi Minh City, Ho Chi Minh City, Vietnam; ^2^Department of Endoscopy, University Medical Center at Ho Chi Minh City, Ho Chi Minh City, Vietnam; ^3^Faculty of Medicine, Nguyen Tat Thanh University, Ho Chi Minh City, Vietnam; ^4^Department of Internal Medicine, Go Cong Regional General Hospital, Gò Công, Vietnam

**Keywords:** gastroesophageal reflux disease, dyspepsia, overlap, endoscopy, Vietnam, functional dyspepsia

## Abstract

**Aim:**

To assess (1) the overlap rate of gastroesophageal reflux disease (GERD) and functional dyspepsia (FD) and (2) the yield of esophagogastroduodenoscopy in patients clinically presenting with FD.

**Materials and Methods:**

Outpatients aged ≥18 years with typical reflux symptoms ≥2 times a week or clinically fulfilling the Rome IV criteria for FD were recruited and underwent esophagogastroduodenoscopy. GERD was classified into non-erosive reflux disease (NERD) and erosive reflux disease (ERD), and FD was classified into epigastric pain syndrome and postprandial distress syndrome. The endoscopic findings that could explain patients’ symptoms were considered clinically significant endoscopic findings. After esophagogastroduodenoscopy, patients were categorized into three groups: GERD-only, FD-only, and GERD-FD overlap.

**Results:**

There were 439 patients with a mean age of 42.3 ± 11.6 years. Ninety-one (20.7%) patients had clinically significant endoscopic findings: 73 (16.6%) reflux esophagitis, 6 (1.4%) Barrett’s esophagus and 14 (3.2%) gastroduodenal ulcers. After excluding gastroduodenal ulcers, the numbers of patients with GERD-only, FD-only, and GERD-FD overlap were 69 (16.2%), 138 (32.5%), and 218 (51.3%), respectively. Postprandial distress syndrome was more prevalent in GERD-FD overlap than in FD-only (72.9 vs. 44.2%, *p* < 0.001). The rates of gastroduodenal ulcers in patients clinically fulfilling the criteria for FD with and without reflux symptoms were 0.6 and 4.7%, respectively (*p* = 0.027).

**Conclusion:**

The GERD-FD overlap was more common than each disorder alone, of which postprandial distress syndrome was significantly prominent. Organic dyspepsia was uncommon in patients clinically fulfilling the Rome IV criteria for FD.

## Introduction

Gastroesophageal reflux disease (GERD) and functional dyspepsia (FD) are the two most common upper gastrointestinal disorders. GERD is generally classified into non-erosive reflux disease (NERD) and erosive reflux disease (ERD), and FD is classified according to the Rome IV criteria into epigastric pain syndrome and postprandial distress syndrome (1, 2). Although there are separate guidelines for managing each disorder, GERD-FD overlap is very common. This can be explained by shared pathophysiology, including delayed gastric emptying and impaired gastric accommodation, between the two disorders (3). Compared to patients suffering from GERD or FD alone, patients with GERD-FD overlap have a much more significant symptom burden and increased medical consultations (4).

Although there have been several studies about this issue, there are still inconsistent findings. The prevalence of GERD-FD overlap varies significantly across populations depending on the studied subjects and diagnostic criteria (3, 5). Few studies have investigated the overlap of GERD with each FD subtype according to the Rome IV criteria. In daily practice, the diagnosis of FD is established mainly based on the clinical ground, but esophagogastroduodenoscopy is essential to rule out organic dyspepsia. Data regarding the yield of esophagogastroduodenoscopy in patients who clinically fulfilled the FD criteria are still limited. This is a clinically significant issue as esophagogastroduodenoscopy is invasive and could be costly in some regions. Vietnam is among the countries with the highest prevalence of *Helicobacter pylori (H. pylori)* infection and *H. pylori*-related gastrointestinal diseases in Southeast Asia (6). GERD with coexisting dyspeptic symptoms has been reported to be very popular, but there have been no data about GERD-FD overlap in this population (7). This study aimed to assess the prevalence of GERD-FD overlap and the yield of esophagogastroduodenoscopy in Vietnamese patients clinically fulfilling the Rome IV criteria for FD. The results of this study would shed further light on the worldwide understanding of this crucial issue.

## Materials and Methods

### Patient Recruitment

Patients aged ≥18 years at the outpatient department of University Medical Center at Ho Chi Minh City, Vietnam, were invited to participate in this study if they had (a) troublesome typical reflux symptoms (heartburn or regurgitation) at least twice a week or (b) dyspeptic symptoms that clinically fulfilled the Rome IV criteria for FD. The exclusion criteria were as follows: (a) prior upper gastrointestinal surgery, (b) prior history of *H. pylori* eradication, (c) prior history or current diagnosis of any of the following: stroke, myocardial infarction, cirrhosis, and malignancy, (d) the use of proton pump inhibitors, H_2_-receptor antagonists, aspirin, non-steroidal anti-inflammatory drugs, or antibiotics within 4 weeks to enrollment, and (e) pregnancy. The study protocol conforms to the ethical guidelines of the 1975 Declaration of Helsinki. This study was approved by the Board of Ethics in Biomedical Research of the University of Medicine and Pharmacy at Ho Chi Minh City, Vietnam (numbered 446/HDDD-DHYD, signed on August 30, 2019). All participants were asked to provide written informed consent.

### Data Collection

Before undergoing esophagogastroduodenoscopy, all participants provided a medical history and were examined by two investigators (QH and CN) to obtain the necessary data according to a predesigned questionnaire. The information in this questionnaire included (a) demographic data (age, sex, body mass index, waist-to-hip ratio, smoking, and alcohol use), (b) upper gastrointestinal symptoms (heartburn, regurgitation, epigastric pain, epigastric burning, early satiety, postprandial fullness, nausea, and vomiting), and (c) alarm features (family history of gastric cancer, unintended weight loss, gastrointestinal bleeding, anemia, dysphagia, odynophagia, and abdominal mass). In this study, smoking was defined as smokers (former or current smoking) or non-smokers, and alcohol consumption was defined as high intake drinkers (>6 drinks per week) or none/low intake drinkers (≤6 drinks per week) (8). The individual items of dyspeptic symptoms necessary to subtype FD according to the Rome IV criteria were also recorded (1).

All endoscopic procedures were performed by five investigators, who were attending physicians of the Department of Endoscopy (QL, DN, NV, ND, and NL), using standard endoscopes (model GIF-XQ160, Olympus Corp., Tokyo, Japan). All endoscopists were blinded to the questionnaire data. The biopsy was obtained at the discretion of the endoscopists. The *H. pylori* infection status was assessed using a local rapid urease test with similar accuracy to PyloriTek^®^ (Serim Research Corp., Elkhart, IN, United States) (9).

### Definitions

Gastroesophageal reflux disease was defined as having typical reflux symptoms at least twice a week or having endoscopic objective evidence of GERD (i.e., reflux esophagitis according to the Los Angeles classification, ulcer, or stricture caused by reflux disease or Barrett’s esophagus) (2). GERD was classified into NERD (i.e., having troublesome reflux symptoms without endoscopic evidence) and ERD (i.e., having endoscopic evidence of GERD with or without reflux symptoms).

FD was defined according to the Rome IV criteria, which include (a) having one or more of the four following bothersome symptoms: postprandial fullness, early satiation, epigastric pain, and epigastric burning; (b) these symptoms fulfilled for the last 3 months with symptom onset and at least 6 months before FD diagnosis; and (c) no evidence of organic, systemic, or metabolic disease that is likely to explain the symptoms on routine investigations (including at esophagogastroduodenoscopy) (1). FD was classified into epigastric pain syndrome and postprandial distress syndrome. Gastroduodenal ulcers and upper gastrointestinal tumors were considered causes of organic dyspepsia and excluded the diagnosis of FD. However, the endoscopic evidence of GERD did not exclude FD, and patients were considered to have GERD-FD overlap.

The endoscopic findings of organic gastrointestinal diseases that could explain the patients’ symptoms were considered clinically significant endoscopic findings. In this study, these findings consisted of reflux esophagitis, stricture, or esophageal ulcer caused by gastroesophageal reflux, Barrett’s esophagus, peptic ulcer diseases, and upper gastrointestinal malignancies.

After undergoing esophagogastroduodenoscopy, patients with gastroduodenal ulcers and upper gastrointestinal malignancy were excluded. The remaining patients were categorized into three groups: GERD only, FD only, and GERD-FD overlap.

### Statistical Analysis

Categorical data are presented as numbers and percentages and were compared using Pearson’s chi-squared test. Quantitative data were tested for normality using the Kolmogorov–Smirnov test. Those with a normal distribution are presented as the mean and standard deviation (*SD*). Those with a non-normal distribution are presented as the median and interquartile range (*IQR*) and were compared using the Mann–Whitney *U* test. Univariate analysis was performed to identify the clinical factors associated with clinically significant endoscopic findings. The factors with *p* values < 0.2 in univariate analysis were used for multiple logistic regression analysis to identify the independent risk factors for clinically significant endoscopic findings. All tests were 2-sided, and a *p* value < 0.05 was considered significant. All statistical analyses were carried out by using the R software (version 4.1.0, Auckland, New Zealand), available from https://cran.r-project.org.

## Results

**FIGURE 1:**
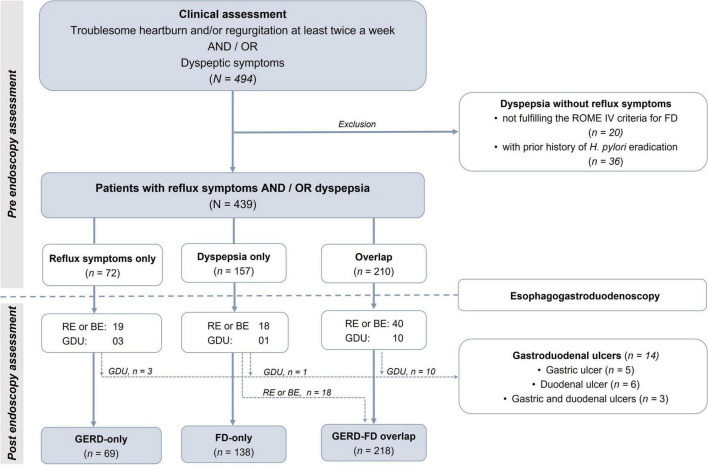
Assessment and classifications of recruited patients before and after endoscopy. FD, functional dyspepsia; GERD, gastroesophageal reflux disease; RE, reflux esophagitis; BR, Barrett’s esophagus; GDU, gastroduodenal ulcer.

A total of 494 patients presenting with typical reflux symptoms or dyspepsia were invited to participate in this study. After clinical assessment, there were 439 patients fulfilling the recruitment criteria (Figure 1). The mean age of the recruited patients was 42.3 (range 19–75), and the male-to-female ratio was 1:1. The demographic and clinical-endoscopic characteristics of these patients are presented in Table 1. The categorizations of recruited patients are presented in Figure 1. After undergoing esophagogastroduodenoscopy, fourteen patients identified with gastroduodenal ulcers were excluded from the analysis. The proportions of GERD only, FD only, and GERD-FD overlap groups were 16.2% (69/425), 32.5% (138/425), and 51.3% (218/425), respectively.

**TABLE 1:** Demographic, clinical, and endoscopic characteristics of recruited patients.

Patients characteristics	
Age (mean ± SD)	42.3 ± 11.6
**Sex, *n (%)***	
Male	218 (49.7)
Female	221 (50.3)
**Body mass index, *n (%)***	
<18.5	55 (12.5)
18.5–22.9	208 (47.4)
23–24.9	87 (19.8)
25–29.9	82 (18.7)
≥30	7 (1.6)
**Smoking, *n (%)***	
Yes	114 (26.0)
No	325 (74.0)
**Alcohol drinking, *n (%)***	
High intake	126 (28.7)
None/low intake	313 (71.3)
***H. pylori*** infection, ***n (%)***	
Yes	150 (34.2)
No	289 (65.8)
**Chief complaints, *n (%)***	
Heartburn	45 (10.3)
Regurgitation	83 (18.9)
Epigastric pain	234 (53.3)
Epigastric burning	14 (3.2)
Early satiety	2 (0.5)
Postprandial fullness	59 (13.4)
Vomiting or nausea	2 (0.5)
**Alarm features, *n (%)***	
Family history of gastric cancer	22 (5.0)
Unintended weight loss	19 (4.3)
Anemia	3 (0.7)
Dysphagia/odynophagia	70 (15.9)
Abdominal mass	0 (0)
Suspected gastrointestinal bleeding	34 (7.7)
**Clinically significant endoscopic findings, *n (%)***	
Reflux esophagitis grade A	62 (14.1)
Reflux esophagitis grade B	11 (2.5)
Barrett’s esophagus	6 (1.4)
Gastroduodenal ulcers	14 (3.2)

There were 287 patients with GERD: 228 (79.4%) with NERD and 59 (20.6%) with ERD. There were 356 patients with FD: 136 (38.2%) with epigastric pain syndrome only, 53 (14.9%) with postprandial distress syndrome only, and 167 (46.9%) with both FD subtypes. The rates of FD in GERD and GERD in FD were 75.9% (218/287) and 61.2% (218/356), respectively. Ninety-one (20.7%) patients had clinically significant endoscopic findings: 73 (16.6%) reflux esophagitis, 6 (1.4%) short-segment Barrett’s esophagus, and 14 (3.2%) gastroduodenal ulcer. There were no gastrointestinal malignancies. In univariate analysis, the clinical factors associated with clinically significant endoscopic findings included advanced age, male sex, body mass index, smoking, and high intake of alcohol (Table 2). However, only advanced age and male sex remained independently associated with clinically significant endoscopic findings in multivariate analysis (Table 3). The rates of gastroduodenal ulcers in patients clinically fulfilling the criteria for FD with and without reflux symptoms were 0.6 and 4.7%, respectively (*p* = 0.027).

**TABLE 2:** Factors associated with clinically significant endoscopic findings: univariate analysis.

	Clinically significant endoscopic findings		*p*
	
	Yes (*n* = 91)	No (*n* = 348)	
Age (mean ± SD)	44.6 ± 11.5	41.8 ± 11.6	0.039
Male, *n (%)*	67 (73.6)	151 (43.4)	<0.001
BMI (mean ± SD)	23.1 ± 3.6	22.1 ± 3.1	0.027
WHR, median (IQR)	0.86 (0.81–0.89)	0.84 (0.80–0.88)	0.027
Smoker, *n (%)*	37 (40.7)	77 (22.1)	<0.001
High intake drinker, *n (%)*	38 (41.8)	88 (25.3)	0.003
Any alarm features, *n (%)*	24 (26.4)	103 (29.6)	0.635
*H. pylori* infection, *n (%)*	25 (27.5)	125 (35.9)	0.165

*BMI, body mass index; SD, standard deviation; WHR, waist-to-hip ratio; IQR, interquartile range.*

**TABLE 3:** Factors associated with clinically significant endoscopic findings: multivariate analysis.

Variable	Adjusted odds ratio	95% confidence interval of odds ratio	*p*
Age	1.03	1.01–1.05	0.017
Male	3.53	1.75–7.12	<0.001
Body mass index	1.09	1.0–1.2	0.059
Waist-to-hip ratio	0.96	0.91–1.01	0.128
Smoker	1.22	0.67–2.24	0.519
High intake drinker	1.09	0.6–1.98	0.771
*H. pylori* infection	0.70	0.41–1.2	0.188

The demographic characteristics of the GERD-FD overlap group in comparison with those of the GERD-only and FD-only groups are presented in Table 4. There was no significant difference between the GERD-FD overlap and the GERD-only groups. However, the median age and waist-to-hip ratio of the GERD-FD overlap group were significantly higher than those of the FD-only group.

**TABLE 4:** Characteristics of patients in the GERD-FD overlap, FD-only, and GERD-only groups.

Characteristics	GERD-FD (*n* = 218)	FD only (*n* = 138)	GERD only (*n* = 69)	*p*_1_	*p*_2_
Age, median (IQR)	43.5 (34–52)	39.0 (31–48.5)	42.0 (33–50)	0.016	0.530
Male, *n* (%)	111 (50.9)	64 (46.4)	34 (49.3)	0.468	0.921
BMI, median (IQR)	22.3 (20.2–24.5)	21.6 (19.7–24.1)	22.9 (19.8–25.0)	0.101	0.651
WHR, median (IQR)	0.84 (0.80–0.88)	0.83 (0.79–0.87)	0.85 (0.82–0.88)	0.013	0.346
Smoker, *n* (%)	58 (26.6)	34 (24.6)	16 (23.2)	0.773	0.684
High intake drinker, *n* (%)	63 (28.9)	36 (26.1)	22 (31.9)	0.649	0.747

*BMI, body mass index; WHR, Waist-to-hip ratio; IQR, interquartile range; p_1_, GERD-FD overlap group vs. FD only group; p_2_, GERD-FD overlap group vs. GERD only group.*

**FIGURE 2:**
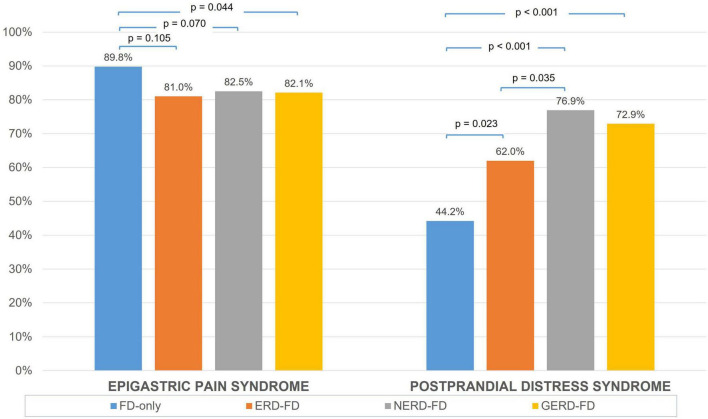
The distribution of epigastric pain syndrome and postprandial distress syndrome across the FD-only and GERD-FD overlap groups. FD, functional dyspepsia; GERD, gastroesophageal reflux disease; ERD, erosive reflux disease; NERD, non-erosive reflux disease.

**FIGURE 3:**
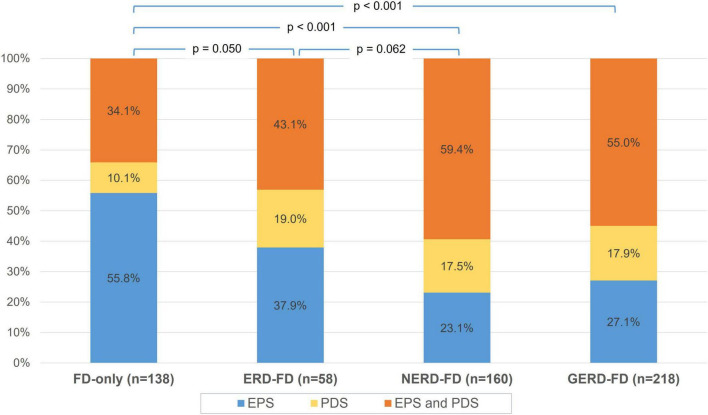
The distribution of epigastric pain syndrome (EPS) and postprandial distress syndrome (PDS) across FD-only and GERD-FD overlap groups. FD, functional dyspepsia; GERD, gastroesophageal reflux disease; ERD, erosive reflux disease; NERD, non-erosive reflux disease.

Regarding the distribution of FD subtypes, postprandial distress syndrome was more prevalent in the GERD-FD overlap group than in the FD-only group (72.9 vs. 44.2%, *p* < 0.001) (Figure 2). Within the GERD-FD overlap group, the rate of postprandial distress syndrome in patients with NERD was also higher than that in patients with ERD (76.9 vs. 62.0%, *p* = 0.035). In contrast, epigastric pain syndrome was less prevalent in the GERD-FD overlap group than in the FD-only group (82.1 vs. 89.8%, *p* = 0.044). The coexistence of both FD subtypes in the GERD-FD overlap group was also significantly higher than that in the FD-only group (*p* < 0.001) (Figure 3).

## Discussion

Our study showed that GERD-FD overlap was very common, accounting for more than half of patients presenting with GERD or FD. Postprandial distress syndrome was more prevalent in the GERD-FD overlap group than in the FD-only group. The yield of esophagogastroduodenoscopy for clinically significant endoscopic findings in these patients was 20.7%, and the rate of organic dyspepsia was very low in dyspeptic patients clinically fulfilling the ROME IV criteria.

It is generally reported that the prevalence of GERD-FD overlap was higher when investigated in the general population compared to the patient population (3, 5). This would be explained by the fact that patients with overlap syndrome had more frequent and severe symptoms and higher scores of anxiety and depression than those suffering from GERD or FD only (10, 11). A recent meta-analysis reported a prevalence of 41.1% (95% *CI*, 29.4–53.9%) FD overlap in patients with GERD and a prevalence of 31.3% (95% *CI*, 19.4–46.2%) GERD overlap in patients with FD (3). The overlap rate in our study was even higher, which could be partially due to the university hospital setting. Future trials on the management of FD and GERD should consider this critical issue in the patient recruiting criteria, as treatment should aim to eliminate the symptoms of both disorders.

Regarding the demographic characteristics, there was no significant difference between the GERD-FD overlap and the GERD-only groups in our study. However, patients in the GERD-FD overlap group were older and had a higher waist-to-hip ratio than those in the FD-only group. A recent study also reported that waist-to-hip ratio but not body mass index was a risk factor for GERD in Vietnamese individuals (7). However, a study in South Korea reported that significantly younger age, female predominance, and lower body mass index were noted in the GERD-FD overlap group than in the GERD-only group (12). Another study in the United States reported no significant differences in age or sex among patients with GERD-FD overlap, GERD only, and dyspepsia only (13). In summary, the demographic characteristics of patients with GERD-FD overlap were inconsistent across studies depending on the investigated populations.

In our study, postprandial distress syndrome was more prevalent in the GERD-FD overlap group than in the FD-only group (Figures 2, 3). The current pathophysiologic understanding of GERD-FD overlap supports our finding. This FD subtype was associated with impaired gastric accommodation, which was demonstrated to significantly increase the occurrence of transient lower esophageal sphincter relaxations and resulted in reflux symptoms (14, 15). However, the distribution of FD subtypes in patients with GERD is inconsistent across studies. A study in South Korea showed that epigastric pain syndrome was more prevalent than postprandial distress syndrome in NERD (68.9 vs. 48.6%, *p* < 0.05) (16). Another study in the United States reported a similar distribution of FD subtypes between the groups of patients with FD-only and GERD-FD overlap groups (17). Other risk factors and pathological mechanisms contribute to this difference. Such data are still sparse, and further studies on other populations are needed.

The rate of clinically significant endoscopic findings in our study was similar to that reported in a previous meta-analysis, in which reflux esophagitis was the most common finding (13.4%), followed by gastroduodenal ulcer (8.0%) (18). In our study, there were no upper gastrointestinal malignancies. A recent endoscopic database of 472,744 Vietnamese patients of the same age as those in our current study who presented with upper gastrointestinal symptoms reported that the rate of upper gastrointestinal malignancy was 0.4% (19). Our study, therefore, suggested that the likelihood of malignancy might be lower in dyspeptic patients clinically fulfilling the Rome IV criteria, even when a significant proportion of them had alarm features. As the majority of clinically significant endoscopic findings were mild-grade reflux esophagitis or short-segment Barrett’s esophagus and the rate of gastroduodenal ulcers was meager, our study suggests that esophagogastroduodenoscopy is unlikely to change the clinical management in most Vietnamese patients presenting with reflux symptoms or clinically fulfilled FD criteria.

There are limited data regarding the yield of esophagogastroduodenoscopy for organic causes of dyspepsia in dyspeptic patients who clinically fulfill the Rome IV criteria for FD. Our study showed a very low rate of gastroduodenal ulcers and no upper gastrointestinal malignancies in such patients. The findings support the recommendation of recent Japanese guidelines on the management of FD, which states that esophagogastroduodenoscopy should be considered individually based on the clinical ground rather than being performed indiscriminately (20). However, these findings should be cautiously interpreted when extrapolated to other populations. For instance, a recent study in China conducted on 381 patients who clinically fulfilled the Rome IV criteria for FD found that the rates of gastroduodenal ulcers and upper gastrointestinal cancer were 4.2 and 2.1%, respectively. This finding suggests that the Rome IV criteria for FD may not be clinically useful to identify FD in Chinese patients. Further studies on other populations regarding this issue are needed.

This study has some limitations. First, this is a single-center, hospital-based study. Therefore, the GERD-FD overlap rate would be higher than that of the general population. Second, the differentiation between true NERD and functional heartburn in our study was impossible, as esophageal pH monitoring and manometry were not performed. However, this limitation may not have a significant impact on our findings, as few patients in our study presented with heartburn, suggesting a low proportion of functional heartburn.

In conclusion, GERD-FD overlap was much more common than each of these two disorders only in Vietnamese outpatients, of whom postprandial distress syndrome was more prevalent. Our study, together with previous studies in this field, demonstrated the high prevalence but different demographic characteristics of GERD-FD overlap across populations. It also showed that esophagogastroduodenoscopy had a low yield of organic dyspepsia in patients clinically fulfilling the Rome IV criteria for FD, even in a population with a high prevalence of *H. pylori* infection and *H. pylori*-related gastrointestinal disorders. In other words, an empiric treatment strategy could be safely initiated in such patients based on their symptoms without the need for esophagogastroduodenoscopy.

## Data Availability Statement

The raw data supporting the conclusions of this article will be made available by the authors, without undue reservation.

## Ethics Statement

The studies involving human participants were reviewed and approved by the Board of Ethics in Biomedical Research of the University of Medicine and Pharmacy at Ho Chi Minh City, Vietnam (numbered 446/HDDD-DHYD, signed on August 30, 2019). All participants provided their written informed consent to participate in this study.

## Author Contributions

DQ designed and supervised the research study, made the first draft, and critically revised and submitted the manuscript. QH, CN, QL, DN, NV, ND, and NL collected the data. DQ and QH analyzed the data. All authors approved the final version of the manuscript.

## Conflict of Interest

The authors declare that the research was conducted in the absence of any commercial or financial relationships that could be construed as a potential conflict of interest.

## Publisher’s Note

All claims expressed in this article are solely those of the authors and do not necessarily represent those of their affiliated organizations, or those of the publisher, the editors and the reviewers. Any product that may be evaluated in this article, or claim that may be made by its manufacturer, is not guaranteed or endorsed by the publisher.
